# COVID-19 severity and age increase the odds of delirium in hospitalized adults with confirmed SARS-CoV-2 infection: a cohort study

**DOI:** 10.1186/s12888-022-03809-2

**Published:** 2022-02-28

**Authors:** Sara C. LaHue, Danielle P. Escueta, Elan L. Guterman, Kanan Patel, Krista L. Harrison, W. John Boscardin, Vanja C. Douglas, John C. Newman

**Affiliations:** 1 Department of Neurology, School of Medicine, University of California, San Francisco, CA USA; 2 Department of Neurology, Weill Institute for Neurosciences, University of California, San Francisco, CA USA; 3 School of Medicine, University of California, San Francisco, CA USA; 4 Division of Geriatrics, Department of Medicine, School of Medicine, University of California, San Francisco, CA USA; 5 Philip R. Lee Institute for Health Policy Studies, University of California, San Francisco, CA USA; 6 Department of Epidemiology & Biostatistics, School of Medicine, University of California, San Francisco, CA USA; 7 Buck Institute for Research On Aging, Novato, CA USA

**Keywords:** Delirium, Encephalopathy, COVID-19, Hospitalization, Restraints, Safety attendants, Hospital discharge, Patient outcomes

## Abstract

**Background:**

Despite recognition of the neurologic and psychiatric complications associated with SARS-CoV-2 infection, the relationship between coronavirus disease 19 (COVID-19) severity on hospital admission and delirium in hospitalized patients is poorly understood. This study sought to measure the association between COVID-19 severity and presence of delirium in both intensive care unit (ICU) and acute care patients by leveraging an existing hospital-wide systematic delirium screening protocol. The secondary analyses included measuring the association between age and presence of delirium, as well as the association between delirium and safety attendant use, restraint use, discharge home, and length of stay.

**Methods:**

In this single center retrospective cohort study, we obtained electronic medical record (EMR) data using the institutional Epic Clarity database to identify all adults diagnosed with COVID-19 and hospitalized for at least 48-h from February 1-July 15, 2020. COVID-19 severity was classified into four groups. These EMR data include twice-daily delirium screenings of all patients using the Nursing Delirium Screening Scale (non-ICU) or CAM-ICU (ICU) per existing hospital-wide protocols.

**Results:**

A total of 99 patients were diagnosed with COVID-19, of whom 44 patients required ICU care and 17 met criteria for severe disease within 24-h of admission. Forty-three patients (43%) met criteria for delirium at any point in their hospitalization. Of patients with delirium, 24 (56%) were 65 years old or younger. After adjustment, patients meeting criteria for the two highest COVID-19 severity groups within 24-h of admission had 7.2 times the odds of having delirium compared to those in the lowest category [adjusted odds ratio (aOR) 7.2; 95% confidence interval (CI) 1.9, 27.4; *P* = 0.003]. Patients > 65 years old had increased odds of delirium compared to those < 45 years old (aOR 8.7; 95% CI 2.2, 33.5; *P* = 0.003). Delirium was associated with increased odds of safety attendant use (aOR 4.5; 95% CI 1.0, 20.7; *P* = 0.050), decreased odds of discharge home (aOR 0.2; 95% CI 0.06, 0.6; *P* = 0.005), and increased length of stay (aOR 7.5; 95% CI 2.0, 13; *P* = 0.008).

**Conclusions:**

While delirium is common in hospitalized patients of all ages with COVID-19, it is especially common in those with severe disease on hospital admission and those who are older. Patients with COVID-19 and delirium, compared to COVID-19 without delirium, are more likely to require safety attendants during hospitalization, less likely to be discharged home, and have a longer length of stay. Individuals with COVID-19, including younger patients, represent an important population to target for delirium screening and management as delirium is associated with important differences in both clinical care and disposition.

## Introduction

Delirium is a life-threatening acute disturbance in mental status affecting more than 2.6 million hospitalized older adults in the United States annually [1]. Delirium is known to be more common in older adults and those requiring intensive care unit (ICU) care [2, 3]. Delirium is also associated with many poor clinical outcomes, including long-term cognitive decline and increased mortality [4, 5]. The severity and inflammatory/vascular pathophysiology of coronavirus disease 19 (COVID-19) suggest that this virus could be particularly deliriogenic, with important implications for the long-term impact of the pandemic [6, 7]. Indeed, delirium is a common complication in patients with COVID-19 and has been shown to be associated with an increased risk of mortality in these patients [8–10]. While the neurological and psychological complications associated with coronavirus disease 19 (COVID-19) are well recognized [11], many studies investigating the impact of COVID-19 on delirium frequency have centered on ICU populations [10, 12] or do not clearly state the criteria for delirium diagnosis [9]. This study sought to identify the association between COVID-19 severity or age with the presence of delirium in hospitalized adults using a systematic in person delirium screening program. In addition, we measured the association between age and presence of delirium, as well as the association between delirium and safety attendant use, restraint use, discharge home, and length of stay in this population of patients with COVID-19.

## Methods

### Study design

For this retrospective cohort study, we obtained electronic medical record (EMR) data using the institutional Epic Clarity database to identify all adult patients hospitalized at the single center for at least 48 h and diagnosed with COVID-19 between February 1, 2020–July 15, 2020. COVID-19 diagnosis was identified using International Classification of Diseases, Tenth Revision code U07.1, which was assigned to all individuals diagnosed with COVID-19 using reverse transcriptase polymerase chain reaction for SARS-CoV-2. COVID-19 infection severity classification was based on World Health Organization criteria within 24 h of admission [13]. COVID-19 severity was classified as follows, from most mild (group 1) to most severe (group 4): hospitalized, no oxygen therapy; oxygen by mask or nasal prongs; non-invasive ventilation or high-flow oxygen; intubation and mechanical ventilation. This study was performed in accordance with the Declaration of Helsinki and was approved by the University of California, San Francisco Institutional Review Board (#20–30,960).

### Delirium metrics

Delirium was defined as a positive Confusion Assessment Method for ICU (CAM-ICU) [14], or a Nursing Delirium Screening Scale (NuDESC) score of 2 or higher [15], at any point during the hospitalization. The presence of delirium was identified using EMR data generated by pre-existing systematic, hospital-wide screening protocols. Our study leveraged the UCSF Delirium Care Pathway [16], which is an interdisciplinary, multicomponent pathway that includes in person twice-daily bedside delirium screening, as well as standardized, evidence-based non-pharmacologic delirium management recommendations.

Under the hospital-wide Delirium Care Pathway, trained bedside nursing staff assess delirium in all hospitalized patients every 12-h shift using tools developed for clinical screening purposes. Delirium in non-ICU acute care patients is assessed using the Nursing Delirium Screening Scale (NuDESC), a validated screening tool for non-ICU hospitalized patients [15]. NuDESC is scored 0–2 via standardized criteria in each of five categories: disorientation, inappropriate behavior, inappropriate communication, hallucinations, and psychomotor retardation. A positive screen is defined by a score of 2 or more. In ICU patients, delirium screenings use the ICU-specific Confusion Assessment Method (CAM-ICU), which assesses acute change or fluctuating course, inattention, disorganized thinking, and level of consciousness [14]. CAM-ICU is also performed in person by the bedside nurse each shift; a positive screen is determined by the bedside nurse using the validated CAM-ICU algorithm [14]. Both NuDESC and CAM-ICU results are entered into the EMR by bedside nursing staff each shift. At least 86% of patients underwent on average two in person delirium screens a day during the study period, which increased to 94% of patients when assessing screening compliance only during full calendar days (e.g., excluding day of admission or discharge), which is similar to prior compliance assessments [16]. The remaining patients underwent on average at least one in person delirium screen a day despite intensive hospital-wide infection control isolation protocols for patients with COVID-19. Of all patients, only two patients had a day without a delirium screen; however, both patients were diagnosed with delirium during their hospitalizations through other screens, so this did not affect our identification of delirium in these patients.

### Outcomes and statistical analysis

The primary analysis was measuring the association between COVID-19 severity and presence of delirium during hospitalization. Secondary analyses included measuring the association between age and presence and delirium; the associations between delirium and safety attendant use, restraint use, discharge to home as opposed to an alternative location, and length of stay were also assessed. In addition to being a hospital-wide quality improvement metric [16], we were particularly interested in restraint use since, due to infection control measures limiting the time staff could safely spend with COVID-19 patients, restraint use may be more common in adults with delirium during the pandemic.

Differences between delirious and non-delirious patients were calculated using Wilcoxon rank-sum tests/t-tests or chi-square tests/Fisher’s exact tests where appropriate. Multivariable logistic regression models assessed the association between initial COVID-19 severity and delirium, as well as the association between delirium and secondary outcomes, adjusting for clinical characteristics. Multivariable linear regression model assessed the association between length of stay and delirium, adjusting for clinical characteristics. To reduce overfitting from limited sample size, COVID-19 severity scores 3 and 4 were combined into one variable, and the variables race and ethnicity were combined into three categories (Table 1). To limit the number of variables in the model, we adjusted only for clinical characteristics that were significant in the univariate analysis: age, race, ethnicity, and COVID-19 severity classification. Due to high correlation and relationship between COVID-19 severity and ICU contact, ICU contact was not included separately in the model. The assumption of linearity for age was evaluated and confirmed. To better illustrate the distribution of delirium within different age groups, the age category was divided into three variables, with model results similar when using age as either a linear or categorized variable. Statistical analysis was performed using Stata 16.1 (StataCorp LLC, College Station, Texas) and SAS version 9.4 (SAS Institute, Inc., Cary, NC).

**Table 1:** Demographics and admission clinical characteristics of hospitalized patients with COVID-19

	No Delirium (*N* = 56)	Delirium (*N* = 43)
Age in Years, Median (IQR)	47.5 (37.5–63)	62 (47–81)
Age in Years, N (%)		
< 45 years	25 (45%)	7 (16%)
45–65 years	22 (39%)	17 (40%)
> 65 years	9 (16%)	19 (44%)
Female Sex, N (%)	16 (29%)	13 (30%)
Race, N (%)		
Asian	8 (14%)	11 (26%)
Black	6 (11%)	5 (12%)
Native Hawaiian or Other Pacific Islander	1 (2%)	1 (2%)
Other	25 (45%)	8 (19%)
Unknown	4 (7%)	1 (2%)
White	12 (21%)	17 (40%)
Ethnicity, N (%)		
Hispanic or Latino	24 (43%)	9 (21%)
Not Hispanic or Latino	29 (52%)	32 (74%)
Unknown	3 (5%)	2 (5%)
Race and Ethnicity combined, N (%)		
Hispanic or Latino	24 (43%)	9 (21%)
White Non-Hispanic	10 (18%)	14 (33%)
Non-White Non-Hispanic	22 (39%)	20 (46%)
Admission Source, N (%)		
Home	40 (71%)	21 (49%)
Skilled Nursing Facility	2 (4%)	5 (12%)
Outside Hospital Transfer	14 (25%)	17 (40%)
Any Intensive Care Unit Contact		
Yes	14 (25%)	30 (70%)
No	42 (75%)	32 (30%)
COVID-19 Severity Classification within 24-Hours of Admission^a^, N (%)		
1–hospitalized, no oxygen therapy	20 (36%)	8 (19%)
2–oxygen by mask or nasal prongs	24 (43%)	18 (42%)
3–non-invasive ventilation or high-flow oxygen	7 (13%)	5 (12%)
4–intubation and mechanical ventilation	5 (9%)	12 (28%)
Hospital length of stay (days), median (q1-q3)	7.5 (4–13)	14 (11–27)

^a^COVID-19 severity was classified as follows: 1 – hospitalized, no oxygen therapy; 2 – oxygen by mask or nasal prongs; 3 – non-invasive ventilation or high-flow oxygen; 4 – intubation and mechanical ventilation

## Results

### Participant demographics and clinical characteristics

A total of 99 patients were diagnosed with COVID-19, of whom 44 patients required ICU care and 17 met criteria for severity scores 3 and 4 combined within 24-h of admission (Table 1). In total, 43 patients (43%) were delirious during hospitalization. Thirty patients (70%) with exposure to ICU-level care were delirious during their hospitalization, compared with 14 (25%) patients with no ICU exposure (p < 0.001). Of patients more than 65 years old, 19 (68%) were delirious, compared with 24 (34%) patients 65 years old and under (*P* = 0.01).

### COVID-19 severity and delirium

Patients with the highest level of COVID-19 severity (groups 3 and 4 combined) within 24 h of admission had 7.2 times the odds of being delirious compared to those with the lowest level of COVID-19 severity (aOR 7.2; 95% CI 1.9, 27.4; *P* = 0.003; Table 2). Delirium was more common in older adults, where patients > 65 years old had 8.7 times the odds of having delirium compared to those < 45 years (aOR 8.7; 95% CI 2.2, 33.5; *P* = 0.003; Table 2).

**Table 2:** Odds of delirium based on COVID-19 severity and age among patients with COVID-19

Characteristic	Unadjusted Odds ratio (95% CI = 95% confidence interval); *P*-value	Adjusted Odds ratio (95% CI = 95% confidence interval); *P*-value
COVID-19 severity*		
1–hospitalized, no oxygen therapy	ref	ref
2–oxygen by mask or nasal prongs vs. 1–hospitalized, no oxygen therapy	1.9 (0.7, 5.2); *p* = 1.0	2.0 (0.6, 6.5); *p* = 0.6*
3/4– ventilation or high-flow oxygen vs. 1–hospitalized, no oxygen therapy	3.5 (1.2, 10.7); *p* = 0.04	7.2 (1.9, 27.4); *p* = 0.003*
Age**		
Age < 45 years	ref	ref
Age 45–65 years	2.8 (0.97, 7.9); *P* = 0.99	2.4 (0.7, 7.5); *P* = 0.64**
Age > 65 years	7.5 (2.4, 23.9); *P* = 0.002	8.7 (2.2, 33.5); *P* = 0.003**

^*^Adjusted for age, race, ethnicity

^**^Adjusted for age, race, ethnicity, and COVID-19 severity classification

### Clinical outcomes by delirium status

The adjusted odds of safety attendant use were 4.5 times higher for those with delirium after adjusting for age, race/ethnicity and COVID-19 severity (aOR 4.5; 95% CI 1.0, 20.7; *P* = 0.050; Table 3). The odds of being discharged to a location other than home were 5 times lower for patients with delirium compared with patients without delirium (aOR 0.2; 95% CI 0.06, 0.6; *P* = 0.005; Table 3). The length of stay was 7.5 days longer for those with delirium (adjusted parameter estimate 7.50; 95% CI 2.0, 13.0; *P* = 0.008; Table 3). Since there were only three patients without delirium with restraints, conclusions regarding the association between delirium and restraint use could not be reached with this small sample size. However, it is worth noting that only 5% of patients without delirium were restrained during hospitalization compared with 58% of patients with delirium.

**Table 3:** Outcomes associated with delirium among patients with COVID-19

Outcome	No Delirium (*N* = 56), n (%) or median days (q1-q3)	Delirium (*N* = 43), n (%) or median days (q1-q3)	Unadjusted Model Result (95% confidence interval); *P*-value	Adjusted* Model Result (95% confidence interval); *P*-value
Safety attendant use odds ratio^	4 (7%)	12 (28%)	5.0 (1.5, 17.0); *P* = 0.009	4.5 (1.0, 20.7); *P* = 0.050
Discharge to home odds ratio^^^	47 (84%)	17 (40%)	0.1 (0.05, 0.3); *P* < 0.0001	0.2 (0.06, 0.6); *P* = 0.005
Length of stay, median days (q1-q3) parameter estimate^#^	7.5 (4–13)	14 (11–27)	10.3 (5.3, 15.3); *P* < 0.0001	7.5 (2.0, 13.0); *P* = 0.008

^*^Adjusted for age, race, ethnicity, and COVID-19 severity classification

^^^Safety attendant use and Discharge to home = Odds ratio (95% confidence interval); *p*-value

^#^Length of stay = parameter estimate (95% Confidence interval); *p*-value

## Discussion

In this study of 99 adults hospitalized with COVID-19 at an academic medical center in Northern California, we found that admission COVID-19 severity was independently associated with increased odds of delirium during hospitalization. This cohort included patients cared for on the acute care medical ward as well as those in the ICU for any portion of their stay, which builds on previously published studies investigating delirium in ICU [10, 12, 17] or emergency department [18] populations in isolation. Importantly, our data suggest that delirium may be underdiagnosed in patients with COVID-19 as our observed delirium frequency was comparable or higher than reported in ICU studies [12, 17] or in older adults upon ED presentation [18]. These data emphasize the urgent need to expand the designation of patient populations at risk for delirium during the COVID-19 pandemic.

We found a high frequency of delirium even in this relatively young cohort, which counters established demographic norms for delirium. While patients with severe COVID-19 were most likely to develop delirium, we found that the frequency of delirium was high even in younger patients. Currently, many hospital-based programs that assess delirium risk and screen for active delirium are targeted to older adults who may benefit the most. Further, studies investigating delirium epidemiology and patient outcomes are often limited to older adult populations; indeed, our recent study investigating clinical outcomes following initiation of our hospital-wide delirium care pathway only included patients who were at least 50 years old [11]. However, here we demonstrated that delirium was also a common complication for younger adults. This suggests that the scales of delirium risk in adults with COVID-19 tip away from age and instead weigh more heavily on the impact of COVID-19 pathophysiology, such as its unique neuroinflammatory cascade serving as a delirium generator. These results suggest that our paradigms of delirium risk in hospitalized adults with COVID-19 should be more inclusive and incorporate rigorous screening for people of all ages.

The primary strength of this study is its ability to leverage the comprehensive, hospital-wide delirium screening program and data collection tools that predated the COVID-19 pandemic. All delirium screens were in person. EMR delirium screening data included all COVID-19 inpatients throughout their stay, including both ICU and non-ICU periods, to comprehensively define delirium rates for the complete hospitalization. We identified delirium cases during a period when COVID-19 hospitalizations were common but did not overwhelm standard screening protocols, though intensive hospital-wide infection control isolation protocols were in effect for patients with COVID-19. Despite these protocols, at least 86% of our study population underwent on average two in person delirium screens a day during the study period, which increased to 94% of patients when assessing screening compliance only during full calendar days. Of all patients, only two patients had a day without a delirium screen; however, both patients were diagnosed with delirium during their hospitalizations using the other screens, so this did not affect our identification of delirium in these patients.

Our EMR-based retrospective study has several limitations. It is possible that the NuDESC screening tool for non-ICU patients underestimated delirium cases, as NuDESC has a high specificity but lower sensitivity compared with more intensive delirium diagnostic methods. Further, we cannot exclude the possibility that part of the association between COVID-19 severity and delirium was due to the use of different screening tools in the ICU (e.g., CAM-ICU), where COVID-19 is more severe, compared with the acute care units (e.g., NuDESC), where COVID-19 is less severe, if the CAM-ICU is more sensitive than the NuDESC. This potential underestimate may be somewhat compensated for by the regularity and frequency of NuDESC screening and would only mean that our already-high measurements of delirium in COVID-19 may be even higher. However, the NuDESC is a well-validated time-efficient delirium screening tool for nursing staff and so was ideal during a time when increased patient load risked compromising established delirium screening protocols. Further, the higher rate of delirium in these patients could be due to factors other than COVID-19. With longer lengths of stay, the patient becomes at risk for delirium due to causes other than the reason for the initial hospitalization, which in our cohort was COVID-19, and because more severe COVID-19 is likely to lead to longer LOS, the higher rate of delirium in these patients could be due to factors other than COVID-19. However, the specificity of the delirium screens used in this study is high [14, 19], so false positives are unlikely. In addition, presence of delirium has been shown to be associated with longer length of stay, and interventions that target delirium reduce length of stay [16]. Because we did not measure the date on which delirium was first diagnosed in each of our patients, we are unable to distinguish between delirium that started on hospital day 2 (and thus was more likely due to COVID-19) and delirium that started on hospital day 30 (and thus was more likely due to a hospital acquired complication). This temporal trend will be an important factor to include in future studies. Additionally, our sample size is relatively small due to relatively low local hospitalization rates at this early phase of the pandemic, though the sample size is similar to previously published studies and sufficiently powered to detect relevant associations.

## Conclusions

Delirium is common in hospitalized patients with COVID-19, especially in those with severe disease on hospital admission, and even in younger patients. Delirium in patients with COVID-19 was associated with several negative outcomes. Individuals with COVID-19, including younger patients, represent an important population to target for delirium screening and management as delirium is associated with important differences in both clinical care and disposition.

## Data Availability

The datasets generated and analyzed during the current study are not publicly available due to them containing information that could compromise participant privacy. The data however are available from the corresponding author (SCL) on reasonable request.

## Abbreviations

aOR: Adjusted Odds Ratio
CI: Confidence Interval
CAM-ICU: Confusion Assessment Method ICU
COVID-19: Coronavirus disease 19
EMR: Electronic medical record
ICU: Intensive Care Unit
NuDESC: Nursing Delirium Screening Scale
OR: Odds Ratio
